# Formulation of Novel Layered Sodium Carboxymethylcellulose Film Wound Dressings with Ibuprofen for Alleviating Wound Pain

**DOI:** 10.1155/2015/892671

**Published:** 2015-05-18

**Authors:** Lenka Vinklárková, Ruta Masteiková, David Vetchý, Petr Doležel, Jurga Bernatonienė

**Affiliations:** ^1^Department of Pharmaceutics, Faculty of Pharmacy, University of Veterinary and Pharmaceutical Sciences Brno, Palackého Třída 1/3, 612 42 Brno, Czech Republic; ^2^Department of Drug Technology and Social Pharmacy, Faculty of Pharmacy, Lithuanian University of Health Sciences, Eiveniu Street 4, LT-50103 Kaunas, Lithuania

## Abstract

Effective assessment and management of wound pain can facilitate both improvements in healing rates and overall quality of life. From a pharmacological perspective, topical application of nonsteroidal anti-inflammatory drugs in the form of film wound dressings may be a good choice. Thus, the aim of this work was to develop novel layered film wound dressings containing ibuprofen based on partially substituted fibrous sodium carboxymethylcellulose (nonwoven textile Hcel NaT). To this end, an innovative solvent casting method using a sequential coating technique has been applied. The concentration of ibuprofen which was incorporated as an acetone solution or as a suspension in a sodium carboxymethylcellulose dispersion was 0.5 mg/cm^2^ and 1.0 mg/cm^2^ of film. Results showed that developed films had adequate mechanical and swelling properties and an advantageous acidic surface pH for wound application. An *in vitro* drug release study implied that layered films retained the drug for a longer period of time and thus could minimize the frequency of changing the dressing. Films with suspended ibuprofen demonstrated higher drug content uniformity and superior *in vitro* drug release characteristics in comparison with ibuprofen incorporation as an acetone solution. Prepared films could be potential wound dressings for the effective treatment of wound pain in low exuding wounds.

## 1. Introduction

The European Wound Management Association (EWMA) Position Document acknowledges that pain is a major issue for patients with acute and chronic wounds [1]. Pain produces stress, which can affect individuals in both psychological and physiological ways and results in delayed wound healing and detrimental effects on quality of life [2]. Therefore, effective assessment and management of wound pain could facilitate an improvement in healing rates and overall quality of life.

Wound-related pain can be temporary (acute) or persistent (chronic) [3]. Acute wound pain can be exacerbated whenever the wound is handled or manipulated: during dressing removal, wound cleansing, or debridement (removing of necrotic tissues). In contrast, persistent (chronic) wound pain is the background symptom that exists at rest and between wound-related procedures.

Wound pain management includes nonpharmacological and pharmacological measures. Multiple pharmacological agents may be used to combat pain. Guidelines for pharmacological wound pain management based on the recommendations by the World Health Organization recommend the use of nonsteroidal anti-inflammatory drugs (NSAIDs) or acetaminophen for patients with mild to moderate pain [3, 4]. NSAIDs provide good pain relief. Moreover, they can positively influence inflammatory processes in the wound, since there is a tendency in chronic wounds for the inflammatory response (an important element in the initial wounding response) to become exaggerated. This results in the increased production of proinflammatory cytokines, reactive oxygen species, and proteolytic enzymes. The chronic wound environment therefore shows sustained inflammation with matrix degradation [5]. Unfortunately, oral use of NSAIDs can lead to serious side effects such as gastrointestinal damage, risk of renal failure, and prolonged bleeding time due to impaired coagulation [6, 7]. For this reason, nonpharmacological strategies and topical agents to achieve optimal wound-related pain management are an attractive solution. Topical agents and correctly selected dressings play a critical role in alleviating wound-related pain [3]. Pain during wound dressing changes or debridement (acute pain) can be substantially reduced using local anesthetics such as lidocaine, tetracaine, or prilocaine applied as a solution, gel, or cream [8, 9]. In the case of chronic wound pain, treating the cause, such as infection or inflammation, as well as optimal wound dressing is of the utmost importance [10]. Wound dressings which are nonadherent and maintain a moist wound environment lead to faster healing and less pain. The pain reduction is attributed to the bathing of the exposed nerve ending in fluid which prevents dehydration of the nerve receptors [10]. Nevertheless, pharmacological measures may be necessary when maintenance of moist wound environment itself is not effective enough for pain reduction or in case of inflammation. For this purpose, topical NSAIDs are an effective option [11].

Topical NSAIDs are formulated for direct application to the painful site and for producing a local pain-relieving effect while avoiding body-wide distribution of the drug at physiologically active levels [12]. Once the drug has reached the site of action, it must be present at a sufficiently high concentration to inhibit cyclooxygenase (COX) enzymes and produce pain relief. Tissue levels of NSAIDs applied topically reach levels high enough to inhibit COX-2 activity [12]. Plasma concentrations found after topical administration, however, are only a fraction of the levels found following oral administration. Recently, an evaluation of the effect of ibuprofen in the form of a foam dressing (Biatain Ibu) on persistent and temporary wound pain underwent clinical trials [13–17]. The ibuprofen foam dressing was shown to consistently relieve wound pain in exuding wounds of various etiologies, irrespective of basal pain intensity. Petersen et al. [18] estimated the ibuprofen foam potential to reduce the need for oral pain killers in two controlled ibuprofen foam trials with the conclusion that local wound pain treatment with ibuprofen foam dressing appears to provide pain relief to the same degree as oral NSAIDs or opioids. Thus, local pain relief by an ibuprofen foam dressing is possible in the most common, painful, exuding, chronic, and acute/traumatic wounds and therefore is a safer alternative for systemic pain treatment [16]. The Biatain Ibu foam dressing may be very useful for patients with painful wounds. However, this dressing has one significant drawback. It would seem that wounds need to have at least a moderate exudate to activate the release of ibuprofen from the dressing; so for those patients with wounds that have low exudate it may not be an option [19]. In such cases, a film made from hydrophilic polymer containing ibuprofen may be a good alternative. The film is thin and needs only a small amount of exudate to activate the release of the drug. For dry wounds, the film may be slightly wetted with normal saline. Moreover, the film is transparent, allowing clinicians to observe a wound's progress without needing to remove it, preserving a moist wound environment [20].

Different polymers may be used to prepare the film. Polyurethane is currently the most used material for such purposes [21]. Polyurethanes, however, are synthetic materials and less friendly on body tissues than materials of a natural origin. Moreover, polyurethane films are intended for the protection of nonexuding wounds and, unless additionally modified, are less suitable for use as drug carriers [20]. For this reason, there have been many studies into how to prepare film wound dressings from natural materials [22–25].

For our experiment, carboxymethylcellulose (CMC), more specifically its sodium salt (sodium carboxymethylcellulose: NaCMC), was chosen because it ranks among the materials with excellent film-forming properties [26]. NaCMC is widely used in pharmaceutical formulations primarily for its ability to increase viscosity [27]. It may also be used for stabilizing emulsions or producing gels. Likewise, the bioadhesive properties of NaCMC are well known. CMC is generally regarded as a nontoxic, nonirritant, and biocompatible material which predestines it for use in food, cosmetic, pharmaceutical, and biomedical applications, including materials for wound care [27]. The suitability and benefits of CMC for application on wounds have been proved by a range of experimental studies. Garrett et al. explored its potential to promote corneal epithelial wound healing [28]. Karami et al. observed its positive effects on wound healing in diabetic male rats [29] and Ramli and Wong observed them on the partial thickness wounds of rats [24]. Currently, NaCMC is used as an absorptive dressing to create conditions for moist wound healing in the field of wound care [27, 30]. The NaCMC dressings on the market do not contain an active substance, with the exception of incorporated silver [30]. NaCMC films have not been employed in wound care yet. Ramli and Wong studied the effect of nonmedicated NaCMC scaffolds on wound healing of rats [24]. Vetchý et al. evaluated the mucoadhesive properties of NaCMC-based films used as dressings to separate the lesion from the environment of the oral cavity [31]. Although CMC films with an active substance as wound dressings have not been investigated yet, there is a whole range of scientific works dealing with the preparation and evaluation of medicated CMC films for other applications, mainly for oral/buccal drug delivery [32–38]. And so the suitability of CMC in the preparation of medicated films has been widely proved. Nevertheless, the application properties of film wound dressings differ quite significantly from those intended for buccal applications. Wound dressings are applied on a much larger surface area than buccal preparations. For this reason, good mechanical properties of medicated CMC films are required. Especially after wetting, they must maintain the cohesiveness that enables them to be easily manipulated and removed without residues. As for other cellulose-based materials, the mechanical properties of CMC-containing films decrease with increasing moisture content [26]. Degree of substitution (DS) also negatively influences the properties of wetted films, since the hydrophilic nature of the film increases with higher DS [39]. On the other hand, the filler, for example, microcrystalline cellulose, if well dispersed in the polymer matrix, usually improves these mechanical properties [26]. So microfibrous NaCMC with relatively low DS (partially carboxymethylated cotton textile) can have a positive effect on the mechanical properties of the film. Partially carboxymethylated cellulose retains its original fibrous nature [40] thus allowing microfibers to act as the filler, whereas the dissolved NaCMC acts as the film-forming agent. The resulting combination can provide improved handling characteristics of the wetted film.

The aim of the presented research was to prepare novel layered films with microfibrous NaCMC and ibuprofen and evaluate their physicochemical properties as well as the influence of the method of ibuprofen incorporation on* in vitro* drug release by modern methods.

## 2. Materials and Methods

The partially substituted (DS 0.35) sodium carboxymethylcellulose in the form of nonwoven textile (Hcel NaT) was supplied by Holzbecher, spol. s r. o., Bleaching & Dyeing Plant in Zlíč (Czech Republic), ibuprofen, macrogol 300, and acetone (all Ph. Eur. grade) were purchased from Fagron (Czech Republic), and the Sanatyl 20 medical grade polyester mesh was purchased from Tylex Letovice, a. s. (Czech Republic). All other chemicals and reagents used in the study were of analytical grade.

### 2.1. Preparation of Films

#### 2.1.1. Preparation of NaCMC Dispersion without and with Ibuprofen

The polymer dispersion was composed of 1% w/w NaCMC and 2% w/w macrogol 300 in purified water. Nonwoven sodium carboxymethylcellulose textile (Hcel NaT) was cut into small pieces and poured over with a solution of macrogol in hot water (80°C). This mixture was then heated to maintain a temperature of 80°C for 3 hours and then left to cool at an ambient temperature for 24 hours. The resulting dispersion was homogenized for 3 min using an ULTRA-TURRAX T 25 dispersing device (IKA Werke Staufen, Germany) at 16,000 rpm. Polymer dispersion with ibuprofen was prepared in the following way. Thoroughly grinded ibuprofen (82.5 mg or 165 mg for one film) was added to the NaCMC dispersion after 24 hours of swelling, and the mixture was homogenized for 8 min using an ULTRA-TURRAX T 25 dispersing device at 16,000 rpm.

#### 2.1.2. Procedure of Preparation

Layered films with or without ibuprofen were prepared with an innovative solvent casting method using a sequential coating technique. This technique involved forming one film and pouring the next layer directly onto the previous one after predrying. The polymer dispersion was casted on an 11 × 15 cm (165 cm^2^) stainless steel plate. Four types of layered films, differing in both concentration and incorporation method of ibuprofen, were prepared (Table 1). The concentration of ibuprofen was 0.5 mg/cm^2^ or 1.0 mg/cm^2^ of film. Ibuprofen was either incorporated between two NaCMC layers or dispersed in the second (upper) layer. The first step was the same for all films. 45 g of NaCMC dispersion were casted on a stainless steel plate, immediately covered with Sanatyl polyester mesh, and then predried in the oven (Heratherm, Germany) at 70°C for 1 hour. The second step was the same for two films (0.5-Ibu-1 and 1.0-Ibu-1): 8.25 g of 1% or 2% ibuprofen solution in acetone were poured on the top of predried film and acetone was evaporated in the hood (approx. 1 hour). Then a layer of 60 g of NaCMC dispersion was poured onto the film and then again predried in the oven at 70°C for 1 hour followed by 24 hours of drying at ambient conditions. For the other two films (0.5-Ibu-2 and 1.0-Ibu-2), 60 g of NaCMC dispersion containing 82.5 mg or 165 mg of ibuprofen were casted, following predrying and drying at the same conditions as previous films. The dried films were peeled from the plates, examined visually for morphological defects (e.g., cracks, shrinking, etc.) which can affect handling, testing, and application as well as aesthetic appearance, and stored in a closed box prior to testing. Films without ibuprofen were made in the same manner for comparison of physical properties (1-blank–4-blank). For the evaluation of morphology of ibuprofen particles, films without Sanatyl polyester mesh were also prepared (0.5-Ibu-1 and 0.5-Ibu-2 without Sanatyl).

### 2.2. Evaluation of Films

#### 2.2.1. Microscopic Properties and Thickness of Films

Microscopic properties of the prepared films were evaluated using an optical microscope (STM-902 ZOOM, Opting, Czech Republic) and a color digital camera (DFW X700, Sony, Japan). The appearance of the films was observed at a magnification factor of 7.5, 20, and 50. Illustrative digital images were taken at the same time.

At the measurement of film thickness, a rectangular sample of the film was vertically secured in a holder, the microscope was focused on the edge of the film, and sample thickness was measured at 5 different places of the film at the points with and without Sanatyl fiber. This was repeated 3 times with each film sample.

#### 2.2.2. Surface pH

Surface pH of the prepared films was evaluated using a WTW pH 3210 SET 2 pH-meter (WTW, Germany) with a flat glass electrode. A moistened pH meter electrode was enclosed in the surface of the film and the value was recorded after stabilization (approximately 30 s). All measurements were taken in triplicate on both sides of the film.

Alterations of the surface pH in the conditions simulating the wound environment were assessed using an artificial wound model (Petri dish, sponge soaked with a physiological buffer solution of pH 7.2). Four cm^2^ (2 × 2 cm) samples of the film were cut and put on the surface of the wound model. The Petri dish was covered with a lid to prevent water evaporation, and surface pH was measured at determined time intervals in triplicate on both sides of the film.

#### 2.2.3. Swelling Property of Films

Swelling properties of the prepared films were measured in a physiological buffer solution of pH 7.2. For these purposes, an artificial wound model was used (Petri dish, sponge soaked with a test liquid). Four cm^2^ (2 × 2 cm) samples of the film were cut and weighed (*W*_*d*_). The sample was placed on the surface of the wound model, the Petri dish was covered with a lid to prevent water evaporation, and swollen films were then weighed at determined time intervals (*W*_*s*_). The degree of swelling Sw in the film was calculated as

(1) $$\begin{array}{c} \text{Sw}=\frac{\left(W_{s}-W_{d}\right)}{W_{d}}. \end{array}$$

#### 2.2.4. Mechanical Properties

A modified method according to Shidhaye et al. was used to evaluate the mechanical properties of the prepared films [41]. A CT3 Texture Analyzer (Brookfield, USA) equipped with a 4.5 kg load cell and TexturePro CT software was used to determine the tensile strength of the prepared films. Film samples (10 × 40 mm) were held between two clamps of a TA-DGA probe positioned at a distance of 2 cm. The lower clamp was stationary and the strips of the film were stretched by the upper clamp moving at a rate of 0.5 mm/s until the strip broke. The work done during this process and the deformation (elongation) of the film at the moment of tearing were also measured. This process was repeated ten times for each film sample.

#### 2.2.5. Drug Content Uniformity

The drug content uniformity test was performed to ensure the uniform distribution of the drug throughout the films. Standard solutions of 0.005, 0.01, 0.015, 0.02, and 0.025% ibuprofen (w/w) were prepared using a physiological buffer solution of pH 7.2 (PBS, pH 7.2). The absorbance values of the standard solutions at 264 nm were measured using a UV spectrophotometer (Lambda 25, Perkin Elmer Instruments, USA), and calibration curves were constructed. Samples (2 × 2 cm) were precisely cut from ten random sites in each film (*n* = 10) and dissolved separately in beakers containing 20 mL of PBS (pH 7.2). Then (after 12 hours) the ibuprofen concentration in the films was determined by measuring the absorbance of the film, relative to the blank (PBS) sample. The average concentrations of ibuprofen (mg/cm^2^) in each sample and SD were then calculated.

#### 2.2.6. *In Vitro* Drug Release

*In vitro* drug release studies on film formulations were performed according to a modified version of Pawar et al.'s method [42]. Specifically, a Franz diffusion cell with an effective diffusion area of 4.73 cm^2^ was used. The receptor compartment of the cell was filled with 20 mL of PBS pH 7.2 as a dissolution medium, while the donor compartment was empty. The prepared film was placed on a thin polyester net between the donor and receptor chambers of the cells. A net was used in order to ensure correct contact between the film's surface and dissolution medium while avoiding immersion. The receptor phase was kept constantly stirred throughout the experiment using magnetic stir bars. The temperature of the receptor compartment was maintained at 32°C using circulating water jackets. At predetermined intervals, 2 mL samples were withdrawn from the receptor phase and replaced with the same amount of PBS pH 7.2 to maintain a constant volume. Drug release was quantified spectrophotometrically at 264 nm and was expressed as cumulative percent released versus time for the 8-hour duration of the study. The kinetics of ibuprofen release from the films were evaluated by determining the best fit of the dissolution data (percentage release versus time) to the Higuchi, Korsmeyer-Peppas, Baker-Lonsdale, Hixson-Crowell, zero order, and first order equations.

### 2.3. Statistical Data Analysis

Data were first analyzed with descriptive statistics and statistical tests (QC Expert, v. 3.2, Trilobyte software) and subsequently with multiple linear regression (MLR) using multiway ANOVA (analysis of variance) with the Unscrambler X program (v. 1.3, Camo software). The design was set for a full factorial with two concentrations of ibuprofen (0.5-Ibu, 1.0-Ibu) and two methods of ibuprofen incorporation (Ibu-1, Ibu-2). Experiments were carried out a minimum of three times, depending on the measured properties. The resulting MLR models were used to identify the influence of process-formulation variables or the effects of their interactions on the measured properties. Film thickness was evaluated by Scheffe's test of pair comparisons in R software, R package: agricolae (v. 1.2-1, Felipe de Mendiburu, 2014).

## 3. Results and Discussion

### 3.1. Formulation and Preparation of Films Containing Ibuprofen

An ideal film dressing must be supple and possess homogenous and smooth surfaces [25]. Transparency is another important property allowing for the wound's assessment without removing the dressing [20]. Films prepared from NaCMC possess all these characteristics and therefore were chosen for the preparation of film dressings with ibuprofen. Partially substituted microfibrous NaCMC (nonwoven textile Hcel NaT) was used in order to increase the mechanical resilience of the films after wetting. This assumption has been proven in our previous experiments, along with the suitability of macrogol 300 as a plasticizer (data have not been published yet). Regardless of the excellent cohesiveness of the wetted films, it was necessary to reinforce them enough to be resistant to surgical devices such as tweezers or in case of application on large areas. For this reason, Sanatyl medical grade polyester mesh was chosen.

In the case of medicated film, an active substance may be dissolved, suspended, or emulsified. Since ibuprofen is poorly water soluble, it is very difficult to achieve its solubility in the film formulation. Thu et al. [25] used cosolvents (propylene glycol and ethanol) for this purpose. In our case, it was impossible because ethanol precipitated the NaCMC from prepared dispersions. The effort to absorb ibuprofen on the Sanatyl led to a high loss of active substance. Thus, ibuprofen was incorporated into the films in solid state in the form of a suspension of previously grinded particles or after crystallization from an acetone solution.

The ibuprofen concentration in the films was expressed as mg/cm^2^ of film. This expression facilitates dosage since wound dressings are applied to a certain surface area. Moreover, it is independent of the weight of the film which may vary considerably, as NaCMC films are hydrophilic with fluctuating moisture content. The same 0.5 mg/cm^2^ concentration of ibuprofen was chosen as in the foam dressing Biatain Ibu [43], which was proved in clinical trials to be effective enough to relieve wound pain [13–17]. Films with double the concentration of 1.0 mg/cm^2^ were prepared for comparison.

### 3.2. Evaluation of Prepared Films

#### 3.2.1. Microscopic Evaluation and Thickness of Films

**Figure 1:**
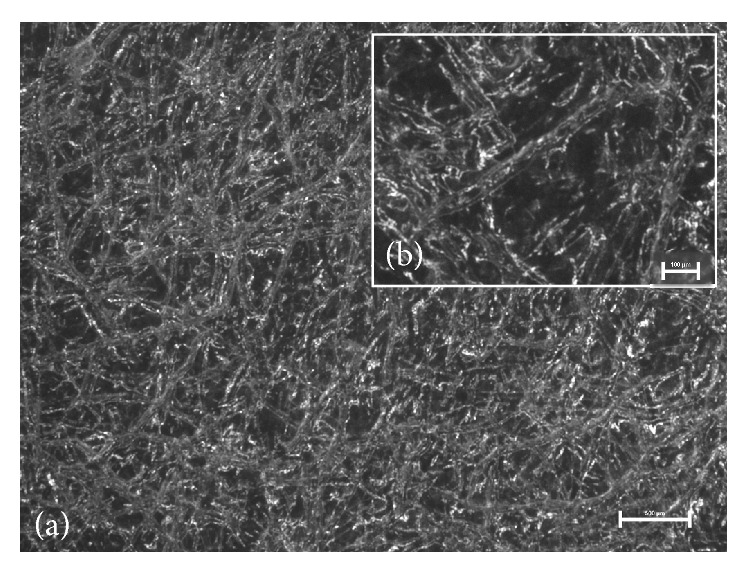
Microscopic appearance of NaCMC film without ibuprofen and Sanatyl: (a) magnification 20x, bar 500 *μ*m; (b) magnification 50x, bar 100 *μ*m.

**Figure 2:**
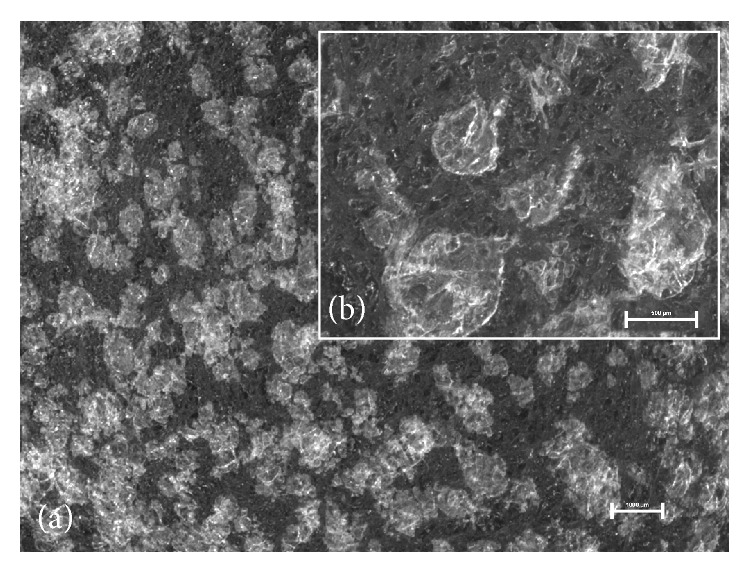
Microscopic appearance of the film with suspended ibuprofen (0.5-Ibu-2 without Sanatyl): (a) magnification 7.5x, bar 1000 *μ*m, (b) magnification 20x, bar 500 *μ*m.

**Figure 3:**
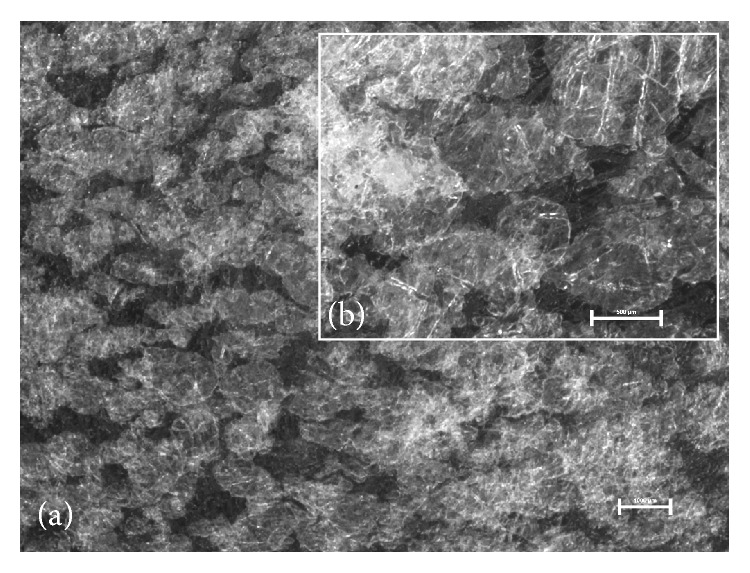
Microscopic appearance of the film with ibuprofen crystallized from acetone solution (0.5-Ibu-1 without Sanatyl): (a) magnification 7.5x, bar 1000 *μ*m, (b) magnification 20x, bar 500 *μ*m.

**Figure 4:**
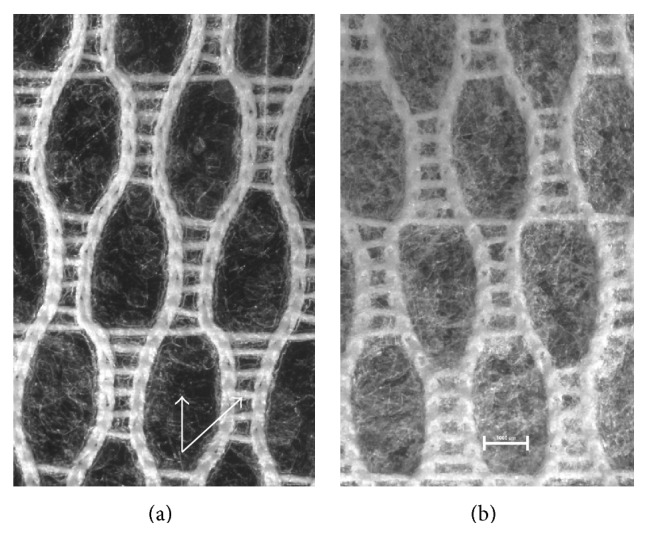
Microscopic appearance of the films with Sanatyl and the same concentration of ibuprofen (magnified 7.5x, bar 1000 *μ*m): (a) film with suspended drug (0.5-Ibu-2), (b) film with drug incorporated as acetone solution (0.5-Ibu-1); arrows mark points where the thickness of films was measured.

Visual examination did not show differences between prepared films—all of them were homogenous and translucent with smooth surface independently of the method of ibuprofen incorporation. Therefore, microscopic evaluation which is capable of imaging inner structure was necessary. Observation of microscopic appearance of prepared films confirmed that partially substituted CMC maintained fibrous nature—digital images showed well-marked microfibrous structures (Figure 1). The films with ibuprofen were found to contain suspended particles of ibuprofen or crystals that formed during acetone evaporation. The suspended particles of ibuprofen seemed smaller (Figure 2) than the crystallized ones (Figure 3) and were distributed in the film more evenly. The crystallized drug was concentrated mostly in the Sanatyl meshes (Figure 4). These findings were important for understanding and explaining results of following evaluations mainly the drug content uniformity and process of ibuprofen release* in vitro*.

Film thickness is an important parameter from the technological point of view. Uniform thickness means correct method of preparation and good assumption to drug content uniformity as well as to regular process of drug release. The thickness of all films with ibuprofen and Sanatyl did not differ significantly and ranged from 152.3 ± 11.7 *μ*m to 165.4 ± 13.9 *μ*m (in points without Sanatyl fiber, *α* = 0.05) or from 340.3 ± 15.3 *μ*m to 361.8 ± 10.3 *μ*m (in points with Sanatyl fiber, *α* = 0.05). The absence of ibuprofen had a negligible effect on the thickness of the film which ranged from 151.6 ± 10.3 *μ*m to 160.9 ± 6.6 *μ*m (in points without Sanatyl fiber) or from 308.5 ± 32.5 *μ*m to 322.1 ± 13.7 *μ*m (in points with Sanatyl fiber). The thickness of films without Sanatyl and without ibuprofen was 186.3 ± 11.6 *μ*m. The results of the film thickness measurement evidenced by low S.D. values showed the sufficient reproducibility of the film preparation method. Higher S.D. values in points with Sanatyl fiber in some samples were owing to material properties of Sanatyl.

#### 3.2.2. Surface pH

Values of surface pH of all prepared films were below 6 (Table 2); that is, surface of the films was acidic. Films without ibuprofen were less acidic and did not differ significantly from each other. Films with the suspended drug had lower pH values on the outside (upper layer) which coincides with the acidic nature of ibuprofen incorporated in the upper layer. By contrast, films prepared using the drug solution in acetone showed lower pH values on the surface intended for contact with the wound, most likely due to a diffusion of acetone solution into the bottom NaCMC layer during evaporation. The null hypothesis of equality of the means (*t*-test) between the sets of films with ibuprofen and without ibuprofen (blank) could not be confirmed, (*P* < 0.01) which points to significant differences in surface pH.

**Figure 5:**
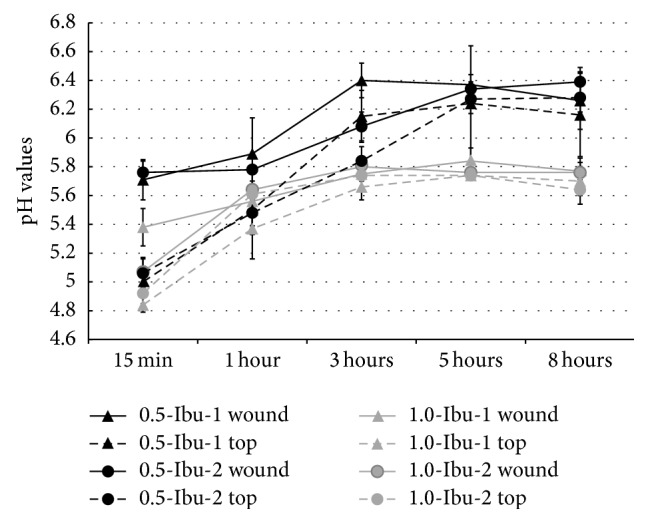
Surface pH of the films with ibuprofen in the conditions simulating a wound environment.

Alterations to the surface pH of the films with ibuprofen during 8 hours in the conditions simulating a wound environment are shown in Figure 5. This evaluation is very important because it reflects the impact of the wound dressing on the wound environment. It is known that pH plays a significant role in wound healing. The pH value within the wound has been shown to affect matrix metalloproteinases (MMPs) activity, tissue inhibitors of MMPs activity, fibroblast activity, keratinocyte proliferation, microbial proliferation, and also immunological responses in a wound [44]. In general, lowering pH has shown to result in an improvement of wound healing. Dressings that directly or indirectly reduce the pH of wound fluid decrease the elevated levels of MMPs which can delay the healing process and may help to prevent infection and improve the antimicrobial activity of some antimicrobials; likewise, oxyhemoglobin releases its oxygen more readily in an acidic environment [44].

Figure 5 demonstrates that all films with ibuprofen retained acidic pH values. Films with the higher concentration of ibuprofen (1.0-Ibu) maintained the lower pH values compared with those containing a reduced amount. In the case of the lower concentration, film 0.5-Ibu-2 was more resistant to PBS than 0.5-Ibu-1. Thus, prepared films, in addition to having an analgesic and anti-inflammatory effect, can also positively influence wound healing rate.

#### 3.2.3. Swelling Property of Films

The swelling behavior of the films is an important property for their practical application. Liquid uptake of the film creates conditions for moist wound healing. It may be affected by several factors such as pH or the presence and character of ions. A physiological buffer solution of pH 7.2 is similar to wound fluid with regard to ion content as well as pH value, and thus determined swelling values of prepared films could adequately reflect those in a real wound.

Films exhibited a mild degree of swelling, indicating moderate holding capacity for the exudate while still maintaining their structural integrity for a reasonable time period. It has been reported that exudate levels in wounds of various etiology differ significantly, as they do in leg ulcers at a range of 0 to 1.2 g/cm^2^/day [43]. In the current study, it was observed that 1 cm^2^ of film with ibuprofen absorbed on average of 0.09 g PBS after 8 hours which indicated that these dressings could be optimal for wounds with low exudate levels.

**Figure 6:**
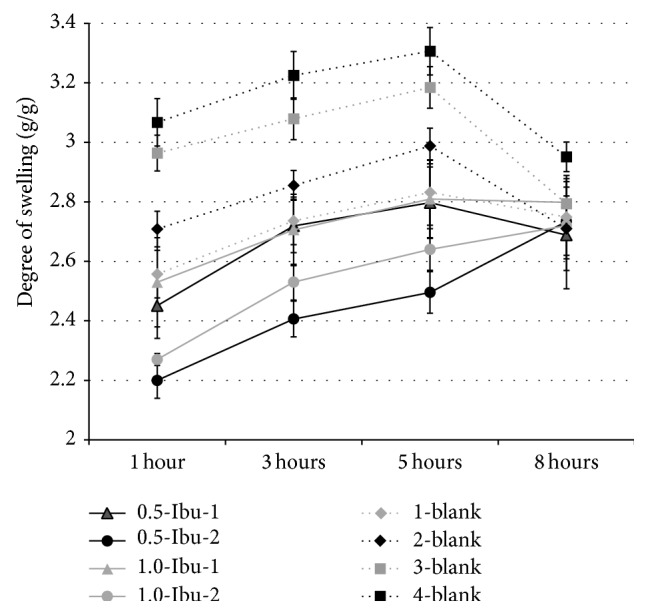
Swelling behavior of prepared films.

Degree of swelling (Sw) was time-dependent and it was in the ascending order 0.5-Ibu-2 < 1.0-Ibu-2 < 0.5-Ibu-1 < 1.0-Ibu-1 < 1-blank < 2-blank < 3-blank < 4-blank (Figure 6). Not surprisingly, the highest degree of swelling was obtained in the case of films without ibuprofen and Sanatyl. Films without ibuprofen and with Sanatyl showed lower swelling values than previous ones due to the minimal swelling capacity of Sanatyl. In the case of all films without ibuprofen, films exposed to acetone during preparation (1-blank, 3-blank) versus others (2-blank, 4-blank) showed a little bit less swelling. Swelling values of all films increased gradually up to 5 hours. The liquid uptake (degree of swelling) of the films without ibuprofen and 0.5-Ibu-1 decreased at the end of the 8-hour observation, possibly due to the partial polymer erosion and dissolving of NaCMC. Film 0.5-Ibu-2 demonstrated the lowest initial degree of swelling, though it continued to swell up until the end of the testing period. 1.0-Ibu-2 demonstrated similar behavior only with a little bit higher liquid uptake. This is most likely due to small particles of suspended ibuprofen in NaCMC dispersion enabling an ion-exchange reaction during preparation with the formation of an insoluble acidic form of CMC. Then, a gradual formation of soluble sodium or potassium salts of the CMC came about after its contact with PBS, which contained monovalent ions, and gradual swelling subsequently ensued.

#### 3.2.4. Mechanical Properties

The mechanical properties of the prepared films are shown in Table 3. Texturometric analysis was used to measure tensile strength (brittleness and hardness of films), deformation/elongation (elasticity and flexibility), and work done during measurement (resilience).

**Figure 7:**
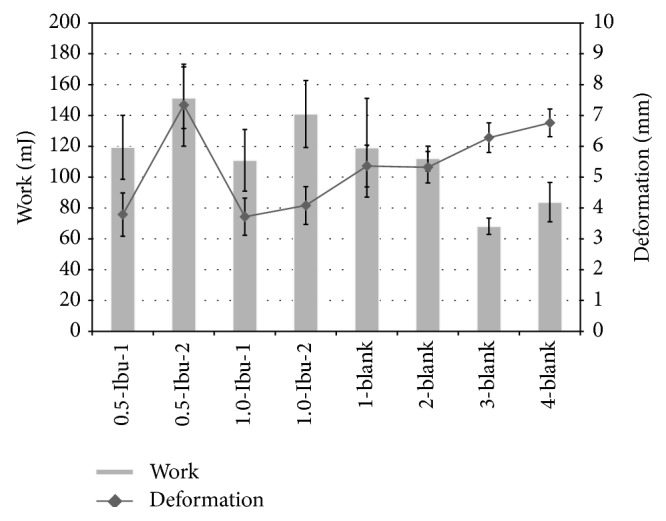
Mechanical properties of films: work done during the process of measurement and deformation/elongation of film at the moment of tearing.

Mechanical properties of the films without ibuprofen were evaluated by Scheffe's test of pair comparisons which confirm a significant effect (*P* < 0.05) of Sanatyl on the work necessary to tear the film and deformation/elongation at the moment of tearing. Films without Sanatyl needed less work and coincidentally elongated more at the moment of tearing (Figure 7).

The influence of process-formulation variables on the mechanical properties of films with ibuprofen was evaluated with MLR regression using ANOVA. The obtained regression models had the following goodness of fit characteristics: *R*-square > 0.7, predicted *R*-square > 0.5, CV < 18%, and the models *P* < 0.01. The impact of ibuprofen concentration and incorporation method was significant (interaction effect *P* < 0.01) in the case of film 0.5-Ibu-2, which needed approximately twice as much tensile strength to tear, and the elongation of film 0.5-Ibu-2 was approximately doubled in comparison with the others (Table 3). This effect may be explained by the formation of an acidic form of CMC during preparation and even dispersion of ibuprofen in the film matrix. The same effect was not observed in the case of 1.0-Ibu-2 probably due to the disturbing influence of the higher content of suspended solid particles on the film matrix. The effect of the method used to incorporate the ibuprofen (Ibu-1 versus Ibu-2) was made most evident in the case of work done (*P* < 0.01), where films with suspended ibuprofen (Ibu-2) yielded values approximately 30 mJ higher than those of Ibu-1 (Table 3, Figure 7).

#### 3.2.5. Drug Content Uniformity

**Figure 8:**
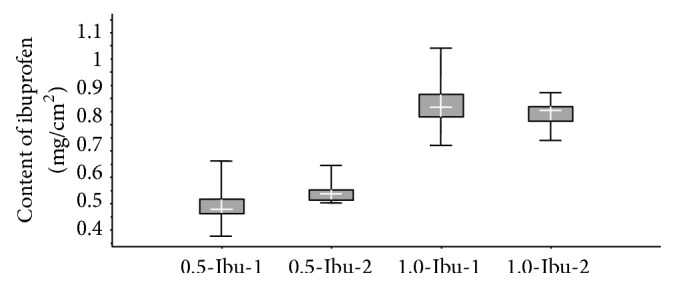
Box diagrams for the drug content uniformity: box encloses 50% of the data and the median as the center of the cross; the whiskers indicate the maximum or minimum value.

The drug content uniformity (*n* = 10) of the layered films is shown in Table 4. Films with suspended ibuprofen (Ibu-2) showed an accurate drug dosage. Ibu-1 films showed wide variation which suggests that the drug was not uniformly distributed. Differences in drug content uniformity are even more noticeable in Figure 8, where the considerable variance is observed in films prepared with an acetone solution (Ibu-1), and, by contrast, the least variance is visible in the case of 0.5-Ibu-2. Crystals of ibuprofen were clustered together during the evaporation of acetone (Ibu-1) (Figure 3), and this was probably the reason why the drug was not evenly dispersed in the film matrix. The negative effect of the clustered ibuprofen crystals on the drug content uniformity in alginate bilayer films was also observed by Thu and Ng [45]. The low variation of values in the case of Ibu-2 indicated that this method of drug incorporation provided reproducible results and could be used to produce a homogeneous drug/polymer matrix system.

#### 3.2.6. *In Vitro* Drug Release

**Figure 9:**
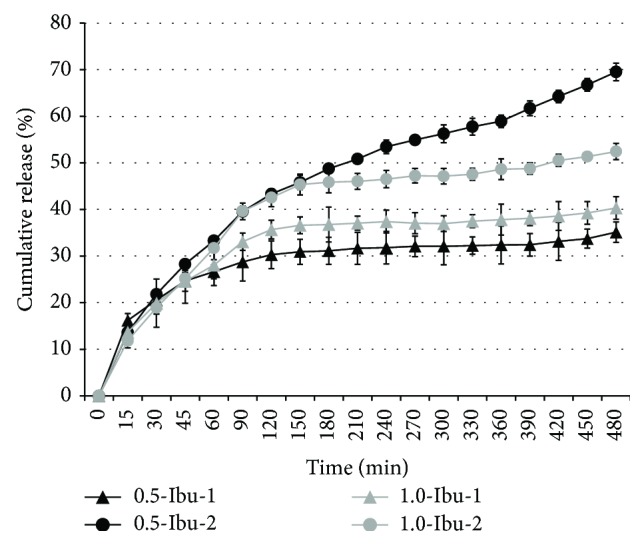
Release of ibuprofen from prepared films.

Generally, ibuprofen release was dependent on the method of its incorporation. When the drug was suspended in an NaCMC dispersion (Ibu-2), about 70% and 50% of ibuprofen were released in the case of 0.5-Ibu-2 and 1.0-Ibu-2, respectively. Incorporation of ibuprofen as an acetone solution retarded drug release, as only about 35% and 40% in the case of 0.5-Ibu-1 and 1.0-Ibu-1 of the drug had been released from the films by the end of 8-hour testing period (Figure 9).

The reason why a larger amount of the released drug in Ibu-2 films was achieved could be that the films with ibuprofen suspended in an NaCMC dispersion (Ibu-2) had smaller particles in comparison with Ibu-1 films, and ibuprofen was released from them more easily. Tang et al. [46] observed a similar impact of ibuprofen crystal size on the drug release profile from chitosan buccal films. Another explanation may be that more soluble and more easily releasable sodium salt of ibuprofen created a parallel with the formation of an acidic form of CMC by ion exchange during film preparation. This explanation is supported also by kinetic models referred to hereinafter.

All prepared films were found intact after the 8-hour dissolution study and exhibited biphasic drug release (Figure 9). This biphasic behavior suggested different mechanisms acting during drug release at each phase. This theory was confirmed by the subsequent analysis in which none of the used kinetic models was able to well describe the obtained dissolution profiles. For this reason, dissolution data were fitted to different kinetic models and the *R* ^2^ values for the six models were calculated for time intervals 0–150 min and 150–480 min (Tables 5 and 6).

Korsmeyer-Peppas, Higuchi, and Baker-Lonsdale models properly described the release of ibuprofen from all films during the first 150 min. In this period, all the films predominantly acted as an insoluble matrix, and the drug was released in two ways, mainly based on Fickian diffusion—by extraction from the matrix into the medium and by leaching through the media which entered into the matrix through the pores. In this stage, the films contained sodium salt of ibuprofen and an acidic form of CMC.

The first order model best described the drug release from all the films in the interval 150–480 min, and Fickian diffusion remained the main mechanism of drug release. Because the drug release was also well described by Hixson-Crowell and zero order models, CMC in the films was probably gradually dissolved due to the gradual formation of soluble sodium or potassium salts of CMC which arose from the acidic form of CMC after contact with PBS containing monovalent ions.

The diffusion process was the main drug release mechanism during both stages of the dissolution study. This finding is supported by Perioli et al.'s statistical evaluation of* in vitro* release of ibuprofen from mucoadhesive patches based on NaCMC for buccal administration [47]. In the earlier stages, the rate of diffusion of the drug from the film polymer was higher than the later stages, which is most likely due to higher concentration gradient. The release of the drug from the polymer matrix occurred slowly after maintaining a certain concentration, and this is a very important observation for designing the controlled drug delivery systems [48]. It was observed that the slow release of drugs from polymeric medicated dressings offers some potential advantages which generally include prolonging the action of the active drug over longer periods of time and allowing continual release from such dosage form and thus improving patient compliance by avoiding the problems brought on by frequent dressing changes [25].

## 4. Conclusions

New film wound dressings with ibuprofen were successfully prepared using an innovative solvent casting method with a sequential coating technique. The films had adequate mechanical and swelling properties and advantageous acidic surface pH for wound application. An* in vitro* drug release study implied that layered films retained the drug for a longer period of time and thus could minimize the frequency of dressing changes. Films with suspended ibuprofen demonstrated better* in vitro* drug release characteristics as well as drug content uniformity. The concentration of suspended ibuprofen which provided the optimal characteristics for a medicated film wound dressing was 0.5 mg/cm^2^ of film.

## Figures and Tables

**Table 1:** Preparation of layered films.

Film	1st step	2nd step	3rd step	4th step
0.5-Ibu-1	NaCMC → Sanatyl → pre-drying	1% Ibu sol. → evaporation	NaCMC	pre-drying and drying
0.5-Ibu-2	NaCMC → Sanatyl → pre-drying	NaCMC with Ibu	—	pre-drying and drying
1.0-Ibu-1	NaCMC → Sanatyl → pre-drying	2% Ibu sol. → evaporation	NaCMC	pre-drying and drying
1.0-Ibu-2	NaCMC → Sanatyl → pre-drying	NaCMC with Ibu	—	pre-drying and drying

1-blank	NaCMC → Sanatyl → pre-drying	acetone → evaporation	NaCMC	pre-drying and drying
2-blank	NaCMC → Sanatyl → pre-drying	NaCMC	—	pre-drying and drying
3-blank	NaCMC → pre-drying	acetone → evaporation	NaCMC	pre-drying and drying
4-blank	NaCMC → pre-drying	NaCMC	—	pre-drying and drying

0.5-Ibu-1 without Sanatyl	NaCMC → pre-drying	1% Ibu sol. → evaporation	NaCMC	pre-drying and drying
0.5-Ibu-2 without Sanatyl	NaCMC → pre-drying	NaCMC with Ibu	—	pre-drying and drying

**Table 2:** Surface pH of the films.

Film	pH of the surface intended for contact with wound	pH of the outside
0.5-Ibu-1	5.17 ± 0.17	5.25 ± 0.26
0.5-Ibu-2	5.18 ± 0.27	5.07 ± 0.26
1.0-Ibu-1	5.12 ± 0.1	5.22 ± 0.19
1.0-Ibu-2	5.27 ± 0.23	4.95 ± 0.23

1-blank	5.42 ± 0.02	5.47 ± 0.09
2-blank	5.62 ± 0.06	5.49 ± 0.08
3-blank	5.67 ± 0.11	5.61 ± 0.08
4-blank	5.61 ± 0.09	5.51 ± 0.06

**Table 3:** Mechanical properties of films.

Formulation	Tensile strength [N]	Deformation/elongation [mm]	Work [mJ]
0.5-Ibu-1	13.35 ± 1.62	3.79 ± 0.71	119.36 ± 20.65
0.5-Ibu-2	22.24 ± 1.62	7.34 ± 1.33	151.47 ± 20.5
1.0-Ibu-1	11.0 ± 0.97	3.72 ± 0.57	110.95 ± 20.04
1.0-Ibu-2	12.89 ± 1.87	4.08 ± 0.61	140.97 ± 21.69

1-blank	17.19 ± 2.36	5.36 ± 0.68	119.11 ± 31.97
2-blank	15.4 ± 1.24	5.32 ± 0.51	112.11 ± 8.02
3-blank	15.87 ± 0.78	6.28 ± 0.48	68.15 ± 5.27
4-blank	17.61 ± 1.39	6.76 ± 0.45	83.8 ± 12.8

**Table 4:** Drug content uniformity in films with ibuprofen.

Formulation	Ibuprofen content (mg/cm^2^)	Number of samples within interval ±10%	Number of samples out of interval ±15%	Number of samples out of interval ±25%
0.5-Ibu-1	0.498 ± 0.091	6	2	2
0.5-Ibu-2	0.542 ± 0.042	9	1	—
1.0-Ibu-1	0.872 ± 0.102	8	2	—
1.0-Ibu-2	0.839 ± 0.056	10	—	—

*n* = 10.

**Table 5:** Kinetic models for the time interval 0–150 min.

Model	Zero order	First order	Higuchi	Hixson-Crowell	Korsmeyer-Peppas		Baker-Lonsdale
Sample	*R* ^2^	*R* ^2^	*R* ^2^	*R* ^2^	*R* ^2^	*n*	*R* ^2^
0.5-Ibu-1	0.828	0.764	0.924	0.828	0.962	0.285	0.883
0.5-Ibu-2	0.905	0.792	0.972	0.905	0.975	0.530	0.968
1.0-Ibu-1	0.887	0.799	0.962	0.887	0.978	0.437	0.944
1.0-Ibu-2	0.913	0.809	0.974	0.913	0.980	0.600	0.966

**Table 6:** Kinetic models for the time interval 150–480 min.

Model	Zero order	First order	Higuchi	Hixson-Crowell	Korsmeyer-Peppas		Baker-Lonsdale
Sample	*R* ^2^	*R* ^2^	*R* ^2^	*R* ^2^	*R* ^2^	*n*	*R* ^2^
0.5-Ibu-1	0.846	0.858	0.812	0.846	0.787	0.083	0.832
0.5-Ibu-2	0.989	0.989	0.979	0.989	0.978	0.335	0.981
1.0-Ibu-1	0.821	0.829	0.770	0.821	0.722	0.066	0.813
1.0-Ibu-2	0.931	0.940	0.893	0.931	0.858	0.116	0.922
